# Qingxin Lianzi Yin Improves Chronic Kidney Disease by Targeting Ferroptosis via the TLR4/HIF‐1α Pathway

**DOI:** 10.1155/mi/7440641

**Published:** 2026-06-05

**Authors:** Binqi Wang, Bo Lin, Danna Zheng, Luxi Cao

**Affiliations:** ^1^ Department of Nephrology, Zhejiang Provincial People’s Hospital (Affiliated People’s Hospital, Hangzhou Medical College), Urology and Nephrology Center, Hangzhou, Zhejiang, China, hospitalstar.com

**Keywords:** chronic kidney disease, ferroptosis, HIF-1α pathway, Qingxin Lianzi Yin, TLR4

## Abstract

**Background:**

Ferroptosis is involved in the pathogenesis of chronic kidney disease (CKD). Qingxin Lianzi Yin (QXLZY) has shown significant advantages in CKD treatment. This investigation intended to explore the impact of QXLZY on ferroptosis in CKD.

**Methods:**

The anti‐CKD effects of QXLZY were evaluated using the 5/6 nephrectomy‐induced rat model and TGF‐β1‐stimulated HK‐2 cells. Oxidative stress and ferroptosis levels were assessed using commercial kits and western blot. The mechanism of QXLZY in CKD treatment was predicted through network pharmacology. TLR4 and HIF‐1α levels were detected by western blot. The role of ferroptosis in CKD treatment by QXLZY was explored using the ferroptosis inducer erastin. HK‐2 cells overexpressing TLR4 were treated with the HIF‐1α inhibitor LW6 to investigate the underlying mechanism. QXLZY components identified through network pharmacology as potentially interacting with TLR4 were validated.

**Results:**

QXLZY effectively alleviated renal injury and inhibited fibrosis and inflammation in CKD models. Moreover, QXLZY markedly suppressed oxidative stress and ferroptosis. However, the anti‐CKD effects of QXLZY were partially counteracted by erastin. TLR4 and HIF‐1α were identified as potential targets through which QXLZY regulates ferroptosis in CKD. Their levels were notably increased in CKD models but were decreased after QXLZY treatment. Overexpression of TLR4 reversed the anti‐CKD effects of QXLZY, and this phenomenon was altered by LW6. Lauric acid (LA) and caprylic acid (CA) were bioactive components within QXLZY regulating the TLR4/HIF‐1α pathway in CKD.

**Conclusion:**

QXLZY inhibits ferroptosis by downregulating the TLR4/HIF‐1α pathway, thereby alleviating renal fibrosis and improving CKD, with LA and CA potentially playing significant roles.

**Summary**

• Qingxin Lianzi Yin (QXLZY) alleviates renal injury and fibrosis in chronic kidney disease (CKD).
• QXLZY inhibits ferroptosis in CKD.
• QXLZY inhibits ferroptosis and improves CKD via the TLR4/HIF‐1α pathway.
• Lauric acid (LA) and caprylic acid (CA) are bioactive components within QXLZY, regulating the TLR4/HIF‐1α pathway.

## 1. Introduction

Chronic kidney disease (CKD) is a progressive disease involving renal structural and functional damage caused by multiple factors [1]. An estimated 10% of adults globally experience CKD, which greatly affects people’s quality of life [1]. CKD therapies are mainly based on RAAS blockade, statin therapy, dietary adjustment, supplementation with compound α‐ketoacid (KA), and control of blood pressure and blood glucose [2, 3]. However, these therapies fail to reverse kidney damage and prevent disease progression. Therefore, it is necessary to further explore effective strategies for CKD treatment.

Ferroptosis is an ROS‐dependent form of cell death, characterized by iron overload and lipid peroxidation, and excessive or defective ferroptosis leads to pathological cell loss and disease development [4]. In healthy individuals, iron‐regulated proteins mediate iron absorption and export by the renal tubules [5]. However, impaired renal function leads to limited iron bioavailability, resulting in iron deposition in the kidneys of CKD patients, thereby inducing ferroptosis and causing renal damage [6]. Previous studies have revealed a marked enhancement in ferroptosis levels in CKD mice, and vitexin intervention inhibited ferroptosis in renal tubular epithelial cells and alleviated CKD [7]. Therefore, targeting ferroptosis in CKD may be a promising therapeutic approach.

Qingxin Lianzi Yin (QXLZY) is a classic prescription in traditional Chinese medicine (TCM) for CKD treatment, originating from the Taiping Huimin Heji Ju Fang (an official Chinese pharmacopoeia of the Song Dynasty) [8]. QXLZY is composed of nine herbs: *Scutellaria baicalensis* (Huangqin), *Ophiopogon japonicus* (Maidong), *Lycium barbarum* cortex (Digupi), *Plantago asiatica* (Cheqianzi), *Glycyrrhiza uralensis* (Zhigancao), *Nelumbo nucifera* (Lianzi), *Poria cocos* (Fuling), *Astragalus membranaceus* (Huangqi), and *Panax ginseng* (Renshen). Studies have revealed that modified QXLZY significantly reduces 24‐h proteinuria levels and alleviates clinical symptoms in patients with IgA nephropathy [9]. Another study shows that QXLZY exerts therapeutic effects in mice with diabetic nephropathy by regulating multiple pathways, such as glycolipid metabolism, oxidative stress, and inflammation [10]. Nevertheless, the influence of QXLZY on ferroptosis in CKD is still unexplored.

TLR4 is a pattern recognition receptor. It initiates a signaling cascade when stimulated, resulting in the production of various inflammatory cytokines and the activation of immune cells [11]. Previous studies have found that TLR4 mutations inhibit interstitial fibrosis and prevent CKD progression in mice with renal fibrosis [12]. Chen et al. [13] discovered that ozone therapy alleviates tubulointerstitial inflammation in CKD rats by inhibiting TLR4. Moreover, research has shown that LPS increases HIF‐1α levels through TLR4 [14]. Zhao et al. [15] demonstrated that Diosmin alleviates ferroptosis and fibrosis in the kidneys by inhibiting HIF‐1α signaling. However, whether QXLZY improves CKD through the TLR4/HIF‐1α pathway remains unexplored.

The current research focus was (1) to evaluate the effects of QXLZY on CKD symptoms and ferroptosis; (2) to investigate how QXLZY regulates ferroptosis in CKD through network pharmacology; and (3) to verify the role of key targets and bioactive components in the treatment of CKD with QXLZY. Regarding pharmacological evaluation, KA, a clinically used therapy for slowing CKD progression, was employed as a positive control to evaluate the therapeutic efficacy of QXLZY in our animal models.

## 2. Methods

### 2.1. Network Pharmacological Analysis

#### 2.1.1. Screening Active Compounds and Potential Targets of QXLZY

The active compounds of QXLZY and their potential targets were obtained from the TCMSP database (https://old.tcmsp-e.com/tcmsp.php) [16]. The selection criteria were set to OB > 30 and DL > 0.18.

#### 2.1.2. Acquisition of CKD‐Related Target Genes and Ferroptosis‐Related Regulatory Factors

The GeneCards (https://www.genecards.org/, relevance score > 5 and protein‐coding), DisGeNET (https://disgenet.com/, GDA score > 0.4), and OMIM (https://www.omim.org/) databases were employed to obtain CKD‐related target genes [17–19]. The ferroptosis gene set was acquired from the FerrDb V2 database (http://www.zhounan.org/ferrdb/current/) [20].

#### 2.1.3. Network Construction

QXLZY targets, CKD targets, and ferroptosis‐related genes were intersected to identify overlapping genes. These genes were uploaded to the STRING database (https://string-db.org/) to construct the PPI network.

#### 2.1.4. GO and KEGG Enrichment Analysis

KEGG and GO enrichment analyses were conducted using the ClusterProfiler R package (Version 4.4.4) [21]. Key KEGG pathways were visualized using the pathview R package (Version 1.46.0).

#### 2.1.5. Differential Expression Analysis

The transcriptome data set GSE66494 for CKD patients was sourced from the GEO database (https://www.ncbi.nlm.nih.gov/geo/), containing 53 CKD samples and 8 normal samples. Differential expression analysis was conducted using the limma R package (Version 3.62.1). Log_2_ transformation and the normalizeBetweenArrays function were employed for data standardization. A design matrix was created using the formula for linear analysis: ~0 + group + batch, and a contrast matrix was created to compare gene expression in CKD patients and controls. The linear model was fitted using lmFit, contrasts.fit, and eBayes. DEGs were identified using topTable, with a threshold of *p* < 0.05 and |log_2_(FC)| > 1.

#### 2.1.6. Molecular Docking

The crystal structure of TLR4 (PDB ID: 3FXI) was retrieved from the RCSB PDB database (https://www.rcsb.org), and the 3D structures of the candidate active ingredients were obtained from the PubChem database. Blind docking was performed using the CB‐Dock2 server, which automatically identifies potential binding sites based on the curviness algorithm and calculates binding affinities using the AutoDock Vina algorithm. A Vina score of ≤−5.0 kcal/mol is considered to indicate potential binding activity, while ≤−7.0 kcal/mol represents strong binding and ≤−9.0 kcal/mol indicates very strong binding.

### 2.2. Experimental Verification

#### 2.2.1. QXLZY Preparation and Quality Control

QXLZY samples (5.0064 g; Tong Ren Tang Co., Ltd., Beijing, China) were extracted with an ethanol–water solution (1:8) under ultrasonic treatment for 30 min and centrifuged to obtain supernatant A [22]. The residue on the filter was mixed with an ethanol–water solution (1:10) under ultrasonic treatment for 30 min, followed by centrifugation to obtain supernatant B. The supernatants A and B were mixed in a volume ratio of 1:1, and the extract was collected after centrifugation. These solutions were passed through a 0.22 µm membrane.

HPLC‐MS was conducted using Agilent 1290 liquid chromatography and 6470 mass spectrometry. Separation was carried out on an Agilent Eclipse Plus C18 RRHD (1.8 μm, 2.1 mm × 50 mm) at 30°C. The mobile phases were 0.1% formic acid aqueous solution (A; Aladdin, W487767) and acetonitrile (B; Sinopharm, 400641692). The flow rate was 0.2 mL/min with a 5 μL injection volume. Gradient elution was conducted according to the conditions given in Table S1. The ionization mode of mass spectrometry and related parameter settings are as follows: m/z range, 100–1000; scanning mode, positive and negative ion modes; gas temperature, 300°C; gas flow, 5.5 L/min; nebulizer, 45 psi; sheath gas temperature, 350°C; sheath gas flow, 11 L/min; capillary, 3500 V; and nozzle voltage, 500 V. HPLC‐MS confirmed the batch consistency of QXLZY, and six main active ingredients (ginsenoside Re, geniposide, baicalin, ammonium glycyrrhizinate, kaempferol, and baicalein) of QXLZY were identified, with retention times of 43.367, 35.900, 12.127, 36.102, 11.675, and 32.075 min, respectively (Figure S1A,B).

#### 2.2.2. Animal Grouping

Thirty Sprague–Dawley rats (male, 7 weeks) were purchased from Beijing HFK Biotechnology Co., Ltd. After 1 week of acclimation, rats were randomly assigned to five groups (*n* = 6): sham, CKD, CKD + QXLZY‐L (7.2 g/kg), CKD + QXLZY‐H (14.4 g/kg, corresponding to a clinically used human equivalent dose of 159 g/day), and KA (1.2 g/kg, a positive control; Lianhuan Health Pharmacy, Jiangsu, China) groups.

The CKD model was constructed using a 5/6 nephrectomy. Rats were anesthetized by an intravenous injection of propofol, and two‐thirds of the upper and lower poles of the left kidney were resected. After 1 week, the entire right kidney was removed under anesthesia. The sham group was subjected to the same operations without kidney removal. After the operation, 0.2 mg/kg buprenorphine was subcutaneously injected into the rats every 12 h for 2 consecutive days. BUN and Scr levels in the serum of rats were measured following 1 week, with their levels being more than twice those of the sham group, indicating successful model establishment. Then, the treatment groups were given the corresponding drugs by gavage every day for 8 weeks. The sham and CKD groups were administered the same volume of normal saline. Following the 8‐week treatment, 24‐h urine samples were collected, and rats were euthanized by inhaling excessive CO_2_. Kidney and serum samples were collected. The 24‐h urinary albumin (UAlb), BUN, and Scr of rats were measured using a fully automatic biochemical analyzer. This study was approved by the Animal Ethical and Welfare Committee of Yanghzou University (Number 202505019), which was carried out in accordance with the U.K. Animals (Scientific Procedures) Act, 1986 and associated guidelines, EU Directive 2010/63/EU for animal experiments.

#### 2.2.3. HE and Masson Staining

The renal tissues of rats were fixed in 4% paraformaldehyde (Beyotime, P0099), embedded in paraffin (Sinopharm, 69019061), and made into 4–7 μm‐thick sections. Sections were stained with the HE staining kit (Beyotime, C0105S) and Masson staining solution (Solarbio, G1340) and then imaged under a microscope (Leica, DM3000).

#### 2.2.4. Immunohistochemistry

Kidney tissues were sectioned at 4 μm thickness and treated with citric acid repair solution (Beyotime, P0083), followed by incubation with 3% H_2_O_2_ (Sinopharm, 10011208). Sections were incubated with the primary antibodies overnight at 4°C, including α‐SMA (1:50; Affinity, AF1032) and Collagen I (1:50; Affinity, AF7001). Subsequently, sections were treated with the secondary antibody (1:1000; Abcam, ab6721) and stained with DAB (Beyotime, P0202). Finally, the sections were counterstained with hematoxylin and imaged using a microscope.

#### 2.2.5. Assessment of Inflammation, Oxidative Stress, and Fe^2+^ Levels

TNF‐α, IL‐1β, and IL‐6 levels were detected using ELISA kits (Mlbio, Shanghai, China). MDA, SOD, and Fe^2+^ levels were detected by commercial kits (Nanjing Jiancheng, Jiangsu, China; Mlbio). These measurements were performed following the manufacturer’s instructions.

#### 2.2.6. Western Blot

Samples were lysed using RIPA buffer (Beyotime, P0013B), and the sample concentration was determined using the BCA method. Protein samples were separated by 10% SDS‐PAGE and transferred onto PVDF membranes (Beyotime, FFP24). The blocked membranes were incubated with the primary antibodies overnight at 4°C, including Collagen I (1:500), α‐SMA (1:500), GPX4 (1:500; Affinity, DF6701), ACSL4 (1:500; Affinity, DF12141), TLR4 (1:500; Thermo Scientific, PA5−23124), and HIF‐1α (1:500; Affinity, AF1009). Membranes were incubated with the secondary antibody for 1 h, and protein bands were visualized using ECL (Applygen, P1000).

#### 2.2.7. Preparation of Drug‐Containing Serum

Rats were randomly divided into normal serum and QXLZY groups. After a 12‐h fast, rats in the QXLZY group were gavaged with QXLZY (14.4 g/kg) for three consecutive days, while those in the normal serum group received the same volume of normal saline. Blood was collected from the heart 2 h after the last intragastric administration under sterile conditions. After standing for 2 h, the supernatant was centrifuged to obtain the drug‐containing serum.

#### 2.2.8. Cell Culture and Intervention

A normal human proximal tubule epithelial cell line, HK‐2 (Procell, CL‐0109, RRID: CVCL_0302), of male origin, was grown in DMEM/F12 (Thermo Scientific, 11320033) supplemented with 10% FBS (Solarbio, S9030) at 37°C with 5% CO_2_. This cell line was authenticated using Short Tandem Repeat analysis, with the matching result of over 80%, and has not been previously reported as misidentified or contaminated. To determine the optimal concentration of QXLZY, cells were treated with TGF‐β1 (10 ng/mL; Baiaolaibo, JN0240) for 24 h with or without different concentrations of QXLZY (5%, 10%, and 20%). To validate the bioactive components from QXLZY identified through network pharmacology analysis as interacting with TLR4, cells were treated with different concentrations of undecanoic acid (UDA; 0, 50, 100, 150, and 200 μM; Yuanye, B25703), lauric acid (LA; 0, 25, 50, 100, and 150 μM; Yuanye, B24119), palmitic acid (PA; 0, 50, 100, 150, and 200 μM; Yuanye, B21705), and caprylic acid (CA; 0, 50, 100, 150, and 200 μM; Yuanye, V34107) for 24 h. Next, their viability was detected using the CCK‐8 assay.

This study included six experiments:

1. Experiment 1: cells were divided into control, TGF‐β1, and TGF‐β1 + QXLZY groups. Cells were treated with TGF‐β1 for 24 h in the presence or absence of 20% QXLZY.
2. Experiment 2: cells were divided into control, TGF‐β1, TGF‐β1 + QXLZY, and TGF‐β1 + QXLZY + erastin groups. Cells were pretreated with 10 μM erastin (a ferroptosis inducer; MCE, HY‐15763) for 3 h prior to treatment with TGF‐β1 and 20% QXLZY for 24 h.
3. Experiment 3: cells were divided into control, TGF‐β1, TGF‐β1 + QXLZY, TGF‐β1 + QXLZY + oe‐NC, and TGF‐β1 + QXLZY + oe‐TLR4 groups. The oe‐TLR4 sequence was designed on the NCBI website. The oe‐TLR4 and oe‐NC lentiviruses were constructed using the third‐generation lentiviral system. HK‐2 cells were plated into a 6‐well plate and grown to 70%–90% confluence. Cells were infected with oe‐TLR4 or oe‐NC lentiviruses for 48 h and then treated with TGF‐β1 and 20% QXLZY for 24 h.
4. Experiment 4: cells were divided into TGF‐β1 + QXLZY + oe‐NC, TGF‐β1 + QXLZY + oe‐TLR4, and TGF‐β1 + QXLZY + oe‐TLR4 + LW6 groups. Cells infected with oe‐TLR4 lentiviruses were pretreated with 10 μM LW6 (a HIF‐1α inhibitor; MCE, HY‐13671) for 3 h, followed by treatment with TGF‐β1 and 20% QXLZY for 24 h.
5. Experiment 5: cells were divided into control, TGF‐β1, TGF‐β1 + UDA, TGF‐β1 + LA, and TGF‐β1 + CA groups. Cells were treated with 10 ng/mL TGF‐β1 for 24 h with or without UDA (100 μM), LA (50 μM), or CA (150 μM), all at noncytotoxic concentrations.
6. Experiment 6: cells were divided into TGF‐β1, TGF‐β1 + LA, TGF‐β1 + CA, TGF‐β1 + LA + oe‐NC, TGF‐β1 + CA + oe‐NC, TGF‐β1 + LA + oe‐TLR4, and TGF‐β1 + CA + oe‐TLR4 groups. Cells were infected with oe‐TLR4 or oe‐NC lentiviruses for 48 h and then treated with 10 ng/mL TGF‐β1 for 24 h with or without LA (50 μM) or CA (150 μM).

#### 2.2.9. CCK‐8 Assay

After grouping treatment, cells were incubated with 10 μL of CCK‐8 solution (Beyotime, C0037) for 2 h, and absorbance at 450 nm was detected using a microplate reader (Wuxi Hiwell Diatek, DR‐200Bs).

#### 2.2.10. Statistical Analysis

Data are expressed as mean ± standard deviation. Statistical analyses were performed on GraphPad 7.0 software using one‐way analysis of variance with the Tukey’s post hoc test. *p* < 0.05 indicated a significant difference.

## 3. Results

### 3.1. Identification of Hub Pathways and Targets of QXLZY in Regulating Ferroptosis in CKD

**Figure 1:**
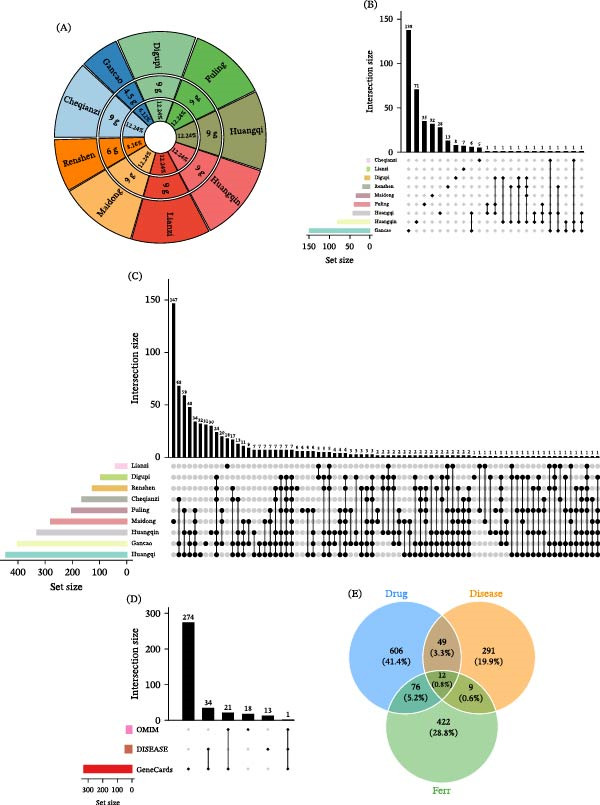
Screening for potential targets of Qingxin Lianzi Yin (QXLZY) regulating ferroptosis in chronic kidney disease (CKD). (A) The herbal composition of QXLZY was obtained from the TCMSP database. Each colored sector represents an individual herb, with its corresponding dosage and percentage indicated. (B) The distribution of active ingredients of traditional Chinese medicine in QXLZY. (C) The distribution of potential targets of QXLZY. (D) The chronic kidney disease (CKD) targets were retrieved from the OMIM, GeneCards and DisGeNET databases. (E) The overlapping genes of CKD targets, QXLZY targets, and ferroptosis‐related genes.

The TCM composition of QXLZY (Huangqin, 9 g; Maidong, 9 g; Digupi, 9 g; Cheqianzi, 9 g; Gancao, 4.5 g; Lianzi, 9 g; Fuling, 9 g; Huangqi, 9 g; and Renshen, 6 g) was obtained from the TCMSP database (Figure 1A), as well as the active compounds and predicted targets. A total of 356 active compounds and 743 potential targets were screened (Figure 1B,C). Moreover, 40, 330, and 48 CKD targets were obtained from the OMIM, GeneCards, and DisGeNET databases, respectively, and a total of 361 were identified after removing duplicates (Figure 1D). The active compounds of QXLZY have been shown to improve CKD by regulating ferroptosis‐related fibrosis [23]. To clarify how QXLZY regulates ferroptosis in CKD, ferroptosis‐related regulatory factors were obtained from the FerrDb V2 database. By intersecting CKD targets, QXLZY targets, and ferroptosis‐related genes, 12 overlapping genes (IL6, IFNG, HNF4A, HMOX1, HIF1A, ESR1, EGFR, CXCL8, COL18A1, TLR4, TGFB1, and NFE2L2) were identified (Figure 1E).

**Figure 3:**
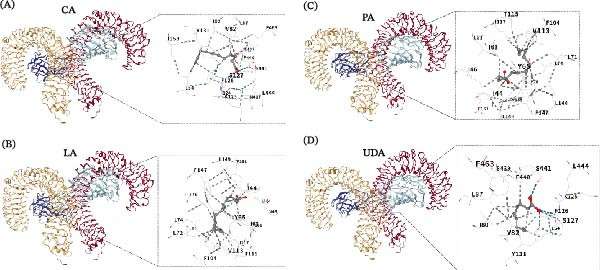
Molecular docking analysis of key active components with TLR4. Molecular docking interactions between TLR4 and CA (A), LA (B), PA (C), and UDA (D), respectively. CA, caprylic acid; LA, lauric acid; PA, palmitic acid; UDA, undecanoic acid.

Enrichment analyses were performed on these 12 genes. GO analysis indicated that these genes were mainly enriched in the positive regulation of chemokine production and miRNA transcription (Figure 2A). KEGG analysis revealed that they were primarily enriched in the HIF‐1 pathway (Figure 2B). Notably, IL6, IFNG, TLR4, RTK, HIF1A, VEGF, and HMOX1 were significantly enriched in the HIF‐1 pathway (Figure 2C). The PPI network of 12 overlapping genes exhibited 12 nodes and 56 edges, indicating strong interactions among them (Figure 2D). The expression of hub genes in CKD patients was assessed, and results revealed that VEGFA expression was markedly downregulated, while EGFR, HIF1A, TLR4, and IFNG expression were upregulated in CKD patients (Figure 2E). A TCM‐component‐target network was constructed. The results suggested that TLR4 might mainly interact with UDA, LA, PA, and CA, and these small molecules primarily originate from Fuling, Huangqi, and Huangqin (Figure 2F). Molecular docking was performed to evaluate the binding characteristics between these components and TLR4. The results showed that UDA (−5.4 kcal/mol), LA (−6.6 kcal/mol), PA (−6.2 kcal/mol), and CA (−5.9 kcal/mol) could all stably bind to TLR4 in a favorable conformation (Figure 3A–D). To confirm the actual presence of these predicted compounds in QXLZY, HPLC‐MS analysis was performed, successfully identifying CA, LA, UDA, and PA, with retention times of 0.801, 0.922, 8.864, and 52.099 min, respectively (Figure S1C).

Figure 2Identification of hub pathways and targets of QXLZY in regulating ferroptosis in CKD. (A) GO enrichment analysis of overlapping genes. (B) KEGG enrichment analysis of overlapping genes. (C) The visualization of the HIF‐1 pathway showing the enriched genes in this pathway. (D) The PPI network of overlapping genes. (E) Differential expression analysis of enriched genes between CKD and normal tissues based on the GSE66494 dataset. (F) The network relationship diagram of “traditional Chinese medicine‐component‐target”.  ^∗^ *p* < 0.05,  ^∗∗^ *p* < 0.01,  ^∗∗∗∗^ *p* < 0.0001 vs. control group.

**Figure:**
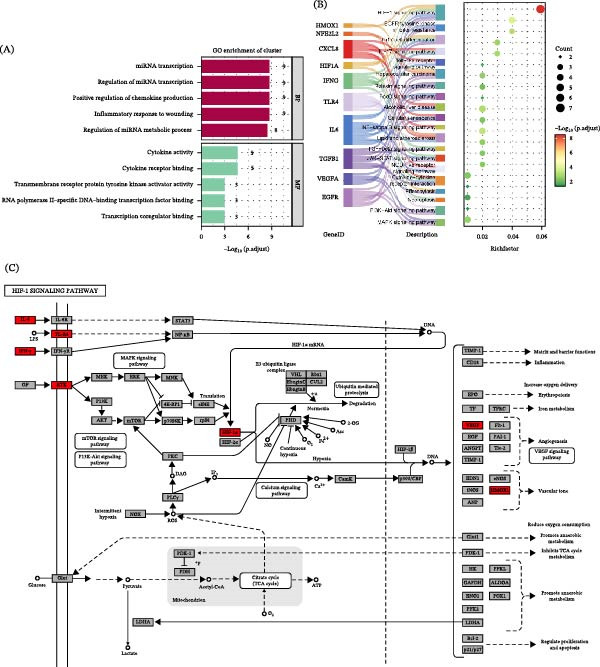

**Figure:**
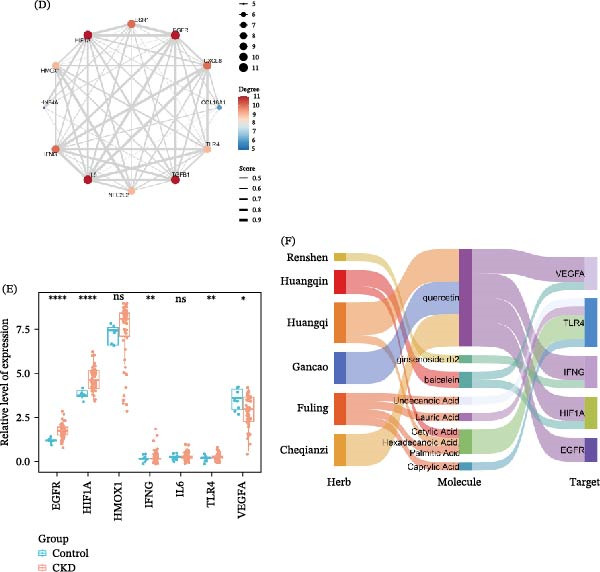

### 3.2. QXLZY Alleviated Renal Injury and Fibrosis in CKD Rats

**Figure 4:**
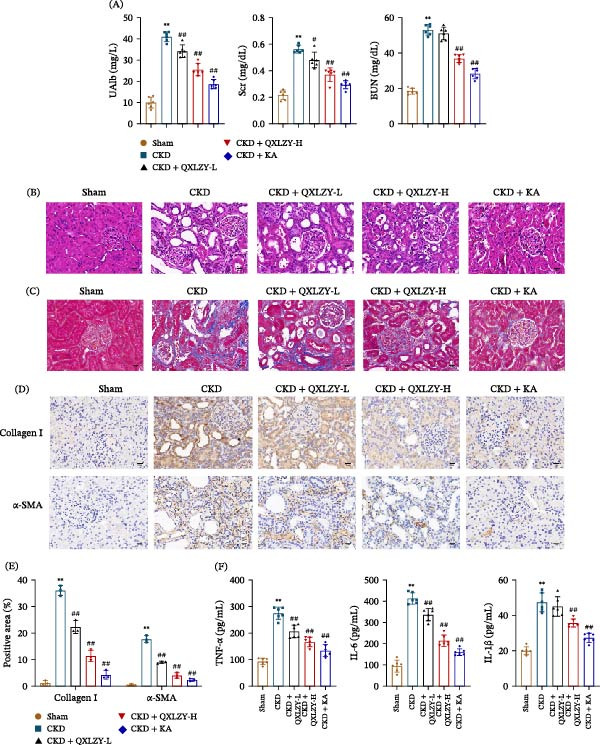
QXLZY alleviated renal injury and fibrosis in CKD rats. (A) The levels of urinary albumin (UAlb), serum creatinine (Scr), and blood urea nitrogen (BUN) were detected by a fully automatic biochemical analyzer. (B) Representative images of HE staining for kidney tissues. Scale bar = 20 μm. (C) Representative images of Masson staining for kidney tissues. Scale bar = 20 μm. (D) The expression of Collagen Ⅰ and α‐SMA in kidney tissues was detected by immunohistochemistry. Scale bar = 20 μm. (E) Quantitative analysis of immunohistochemical results. (F) The levels of TNF‐α, IL‐6, and IL‐1β in kidney tissues were measured using ELISA kits.  ^∗∗^ *p* < 0.01 vs. sham group; #*p* < 0.05, ##*p* < 0.01 vs. CKD group.

Biochemical parameters in rats were detected. UAlb, Scr, and BUN levels in CKD rats were notably increased compared to those in sham rats (Figure 4A). HE staining exhibited normal glomerular and tubular epithelial cells in sham rats, while glomerular injury and atrophy of tubular epithelial cells were observed in CKD rats (Figure 4B). Compared to the sham group, the CKD group showed marked collagen deposition accompanied by elevated Collagen I and α‐SMA expression (Figure 4C–E). Additionally, TNF‐α, IL‐6, and IL‐1β levels were notably increased in CKD rats compared to those in sham rats (Figure 4F). The CKD model was successfully established. Both QXLZY and KA alleviated renal pathological injury and collagen deposition and reduced UAlb, Scr, BUN, Collagen I, α‐SMA, TNF‐α, IL‐6, and IL‐1β levels in CKD rats (Figure 4A–F), indicating that QXLZY ameliorated renal injury and fibrosis in CKD.

### 3.3. QXLZY Inhibited Ferroptosis and Suppressed TLR4 and HIF‐1α Expression in CKD Rats

**Figure 5:**
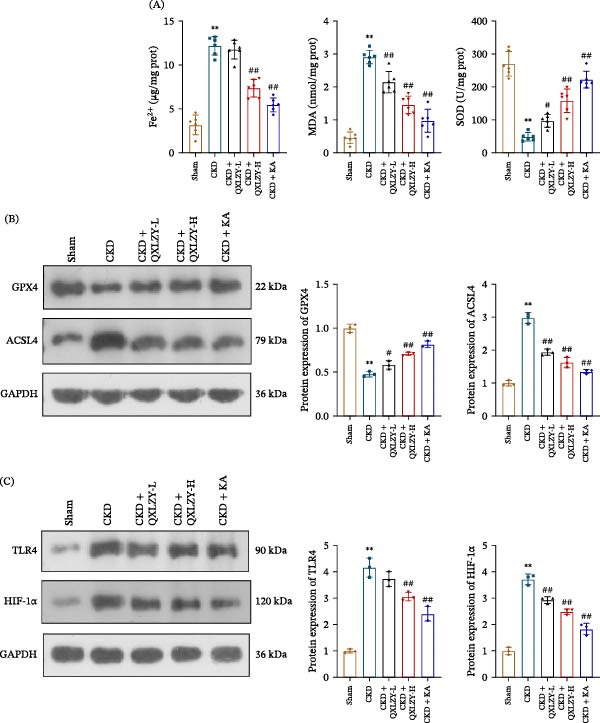
QXLZY inhibited ferroptosis and suppressed TLR4 and HIF‐1α expression in CKD rats. (A) The levels of Fe^2+^, MDA, and SOD in kidney tissues were measured using commercial kits. (B) The levels of GPX4 and ACSL4 in kidney tissues were measured by western blot. (C) The levels of TLR4 and HIF‐1α in kidney tissues were measured by western blot.  ^∗∗^ *p* < 0.01 vs. sham group; #*p* < 0.05, ##*p* < 0.01 vs. CKD group.

Fe^2+^ and MDA levels were significantly increased, whereas SOD levels were decreased in CKD rats compared to sham rats, and these changes were reversed after KA or high‐dose QXLZY treatment (Figure 5A). Moreover, GPX4 levels were notably decreased in CKD rats, while ACSL4 showed an opposite trend, and their levels were reversed by QXLZY or KA (Figure 5B). To confirm the role of TLR4 and HIF‐1α in the treatment of CKD with QXLZY, their levels were detected. The results indicated that TLR4 and HIF‐1α levels were notably increased in the CKD group compared to the sham group, while both KA and high‐dose QXLZY markedly reduced their levels in the CKD group (Figure 5C). QXLZY inhibited ferroptosis and downregulated TLR4 and HIF‐1α expression in CKD.

### 3.4. QXLZY Inhibited Fibrosis and Ferroptosis and Decreased TLR4 and HIF‐1α Expression in TGF‐β1‐Treated HK‐2 Cells

**Figure 6:**
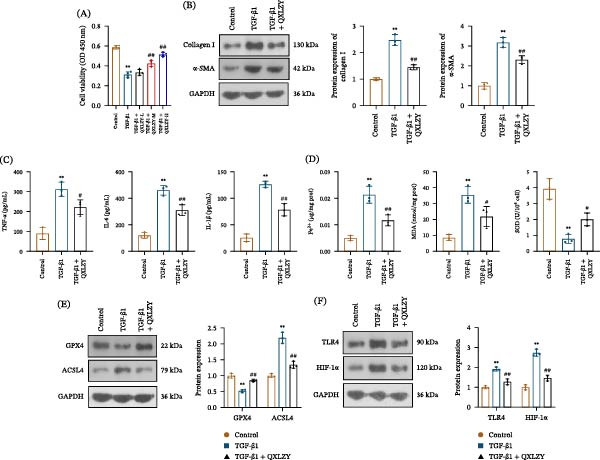
QXLZY inhibited fibrosis and ferroptosis and decreased TLR4 and HIF‐1α expression in TGF‐β1‐treated HK‐2 cells. (A) Cell viability was detected by CCK‐8 assay. (B) The levels of Collagen Ⅰ and α‐SMA were measured by western blot. (C) The levels of TNF‐α, IL‐6, and IL‐1β were measured using ELISA kits. (D) The levels of Fe^2+^, MDA, and SOD were measured using commercial kits. (E) The levels of GPX4 and ACSL4 were measured by western blot. (F) The levels of TLR4 and HIF‐1α were measured by western blot.  ^∗∗^ *p* < 0.01 vs. control group; #*p* < 0.05, ##*p* < 0.01 vs. TGF‐β1 group.

HK‐2 cells were treated with TGF‐β1 with or without different concentrations of QXLZY. The cell viability was markedly decreased after TGF‐β1 stimulation and was partially restored by medium or high concentrations of QXLZY, with the high concentration showing the most significant effect (Figure 6A). Therefore, high‐concentration QXLZY was selected for subsequent experiments. In the TGF‐β1 group, Collagen I, α‐SMA, TNF‐α, IL‐6, and IL‐1β levels were significantly increased compared to the control group, while QXLZY markedly reduced their levels (Figure 6B, C). Furthermore, TGF‐β1 stimulation significantly increased Fe^2+^ and MDA levels and reduced SOD levels in cells, whereas QXLZY reversed this phenomenon (Figure 6D). Additionally, TGF‐β1 significantly increased ACSL4 levels but decreased GPX4 levels in HK‐2 cells, which were altered by QXLZY (Figure 6E). Moreover, TGF‐β1 significantly increased TLR4 and HIF‐1α levels in HK‐2 cells, which were reversed following QXLZY intervention (Figure 6F). QXLZY inhibited fibrosis and ferroptosis and downregulated TLR4 and HIF‐1α expression in an in vitro CKD model.

### 3.5. QXLZY Alleviated Fibrosis and Inflammation in TGF‐β1‐Treated HK‐2 Cells by Inhibiting Ferroptosis

**Figure 7:**
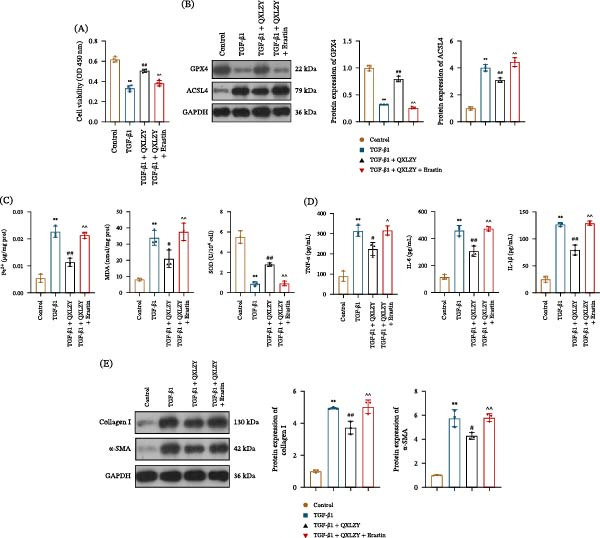
QXLZY alleviated fibrosis and inflammation in TGF‐β1‐treated HK‐2 cells by inhibiting ferroptosis. (A) Cell viability was detected by CCK‐8 assay. (B) The levels of GPX4 and ACSL4 were measured by western blot. (C) The levels of Fe^2+^, MDA, and SOD were measured using commercial kits. (D) The levels of TNF‐α, IL‐6, and IL‐1β were measured using ELISA kits. (E) The levels of Collagen Ⅰ and α‐SMA were measured by western blot.  ^∗∗^ *p* < 0.01 vs. control group; #*p* < 0.05, ##*p* < 0.01 vs. TGF‐β1 group; ^*p* < 0.05, ^^*p* < 0.01 vs. TGF‐β1 + QXLZY group.

To investigate the role of ferroptosis in the treatment of CKD with QXLZY, HK‐2 cells were pretreated with the ferroptosis inducer erastin before treatment with TGF‐β1 and QXLZY. QXLZY partially restored the viability of HK‐2 cells treated with TGF‐β1, but this effect was reversed by erastin intervention (Figure 7A). Compared with the TGF‐β1 group, GPX4 and SOD levels were significantly increased in the TGF‐β1 + QXLZY group, whereas ACSL4, Fe^2+^, and MDA levels showed the opposite trend (Figure 7B, C). However, erastin treatment altered this phenomenon. Moreover, QXLZY reduced the levels of inflammatory factors (TNF‐α, IL‐6, and IL‐1β) and fibrosis markers (Collagen I and α‐SMA) in TGF‐β1‐treated cells, and these effects were partially counteracted by erastin (Figure 7D, E).

### 3.6. QXLZY Suppressed Ferroptosis in CKD In Vitro by Inhibiting TLR4/HIF‐1 α Pathway

**Figure 8:**
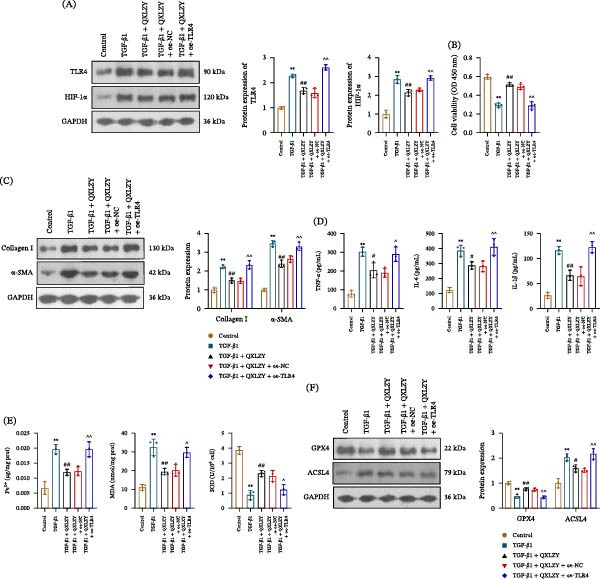
QXLZY suppressed ferroptosis in CKD in vitro by downregulating TLR4 expression. (A) The levels of TLR4 and HIF‐1 α were measured by western blot. (B) The cell viability was detected by CCK‐8 assay. (C) The levels of Collagen Ⅰ and α‐SMA were measured by western blot. (D) The levels of TNF‐α, IL‐6, and IL‐1β were measured using ELISA kits. (E) The levels of Fe^2+^, MDA, and SOD were measured using commercial kits. (F) The levels of GPX4 and ACSL4 were measured by western blot.  ^∗∗^ *p* < 0.01 vs. control group; #*p* < 0.05, ##*p* < 0.01 vs. TGF‐β1 group; ^*p* < 0.05, ^^*p* < 0.01 vs. TGF‐β1 + QXLZY + oe‐NC group.

To further explore the mechanism of QXLZY in treating CKD, TLR4 was overexpressed in HK‐2 cells (Figure 8A). Interestingly, HIF‐1α expression was increased after overexpression of TLR4, suggesting that HIF‐1α might be a downstream target of TLR4 (Figure 8A). QXLZY significantly increased cell viability in the TGF‐β1 group, which was reversed by overexpression of TLR4 (Figure 8B). Furthermore, QXLZY markedly reduced the levels of fibrosis and inflammatory markers in TGF‐β1‐stimulated cells, while overexpression of TLR4 altered the effects of QXLZY (Figure 8C, D). The results indicated that QXLZY notably reduced Fe^2+^, MDA, and ACSL4 levels, and increased SOD and GPX4 levels in the TGF‐β1 group, which were reversed by overexpression of TLR4 (Figure 8E, F). QXLZY inhibited ferroptosis and fibrosis in CKD by downregulating TLR4.

To further elucidate the role of HIF‐1α in the treatment of CKD with QXLZY, the HIF‐1α inhibitor LW6 was employed to treat HK‐2 cells. Overexpression of TLR4 significantly increased HIF‐1α expression in the TGF‐β1 + QXLZY + oe‐NC group, which was inhibited by LW6 (Figure 9A). Moreover, overexpression of TLR4 significantly reduced cell viability and increased the levels of fibrosis and inflammatory markers in the TGF‐β1 + QXLZY + oe‐NC group, while LW6 altered the effects of overexpression of TLR4 (Figure 9B–D). Furthermore, overexpression of TLR4 notably increased Fe^2+^, MDA, and ACSL4 levels and decreased SOD and GPX4 levels, but LW6 reversed this phenomenon (Figure 9E, F). QXLZY alleviated ferroptosis by inhibiting the TLR4/HIF‐1α pathway, thereby ameliorating CKD.

Figure 9QXLZY suppressed ferroptosis in CKD in vitro by inhibiting TLR4/HIF‐1α pathway. (A) The levels of TLR4 and HIF‐1α were measured by western blot. (B) The cell viability was detected by CCK‐8 assay. (C) The levels of Collagen Ⅰ and α‐SMA were measured by western blot. (D) The levels of TNF‐α, IL‐6, and IL‐1β were measured using ELISA kits. (E) The levels of Fe^2+^, MDA, and SOD were measured using commercial kits. (F) The levels of GPX4 and ACSL4 were measured by western blot.  ^∗∗^ *p* < 0.01 vs. TGF‐β1 + QXLZY + oe‐NC group; #*p* < 0.05, ##*p* < 0.01 vs. TGF‐β1 + QXLZY + oe‐TLR4 group.

**Figure:**
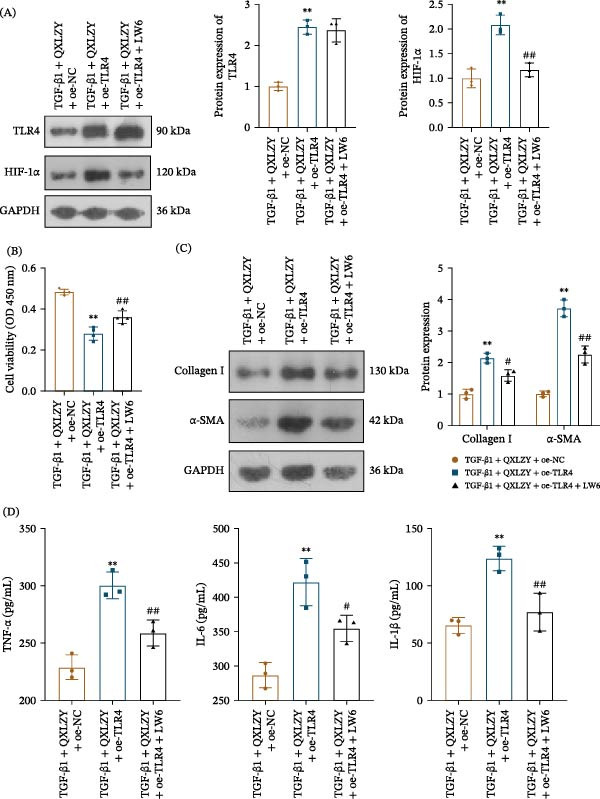

**Figure:**
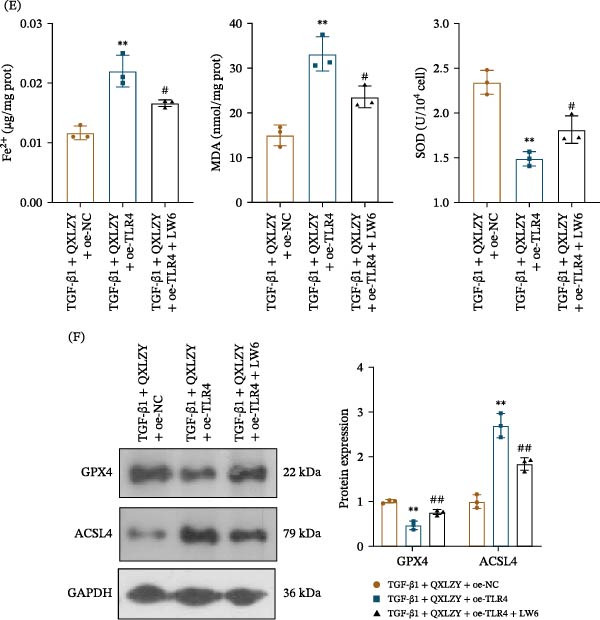

### 3.7. LA and CA Were Bioactive Components of QXLZY that Regulate the TLR4/HIF‐1α Pathway in CKD

**Figure 10:**
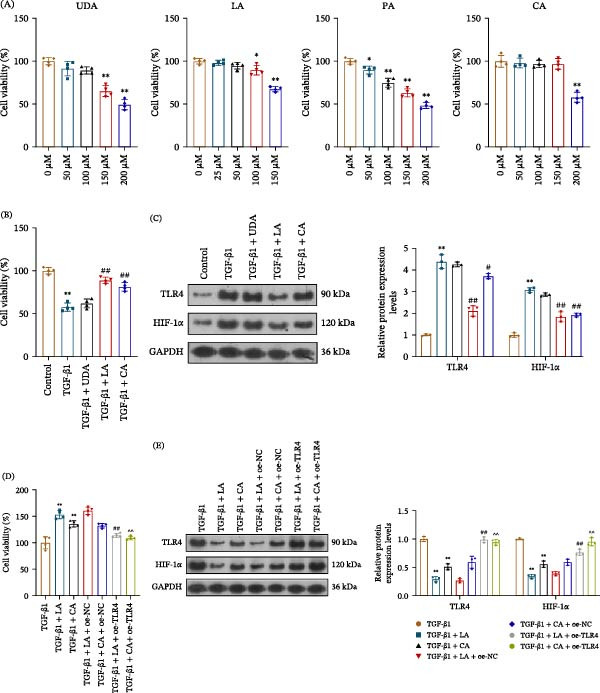
LA and CA were key QXLZY components regulating the TLR4/HIF‐1α pathway in CKD. (A) HK‐2 cells were treated with different concentrations of UDA, LA, PA, and CA for 24 h, and cell viability was evaluated using CCK‐8 assay.  ^∗^ *p* < 0.05,  ^∗∗^ *p* < 0.01 vs. 0 μM group. (B) HK‐2 cells were treated with or without 10 ng/mL TGF‐β1 for 24 h in the presence or absence of UDA (100 μM), LA (50 μM), or CA (150 μM). Cell viability was measured using CCK‐8 assay. (C) The levels of TLR4 and HIF‐1α were measured by western blot.  ^∗∗^ *p* < 0.01 vs. control group; #*p* < 0.05, ##*p* < 0.01 vs. TGF‐β1 group. (D) HK‐2 cells were infected with oe‐NC or oe‐TLR4 lentiviruses for 48 h, and then treated with 10 ng/mL TGF‐β1 for 24 h in the presence or absence of LA (50 μM) or CA (150 μM). Cell viability was measured using CCK‐8 assay. (E) The levels of TLR4 and HIF‐1α were measured by western blot.  ^∗∗^ *p* < 0.01 vs. TGF‐β1 group; ##*p* < 0.01 vs. TGF‐β1 + LA + oe‐NC group; ^^*p* < 0.01 vs. TGF‐β1 + CA + oe‐NC group.

To clarify the bioactive compounds of QXLZY that regulate the TLR4/HIF‐1α pathway in CKD, we conducted in vitro experiments on the four compounds (UDA, LA, PA, and CA) identified by network pharmacology that might interact with TLR4. The CCK‐8 assay demonstrated that all tested concentrations of PA significantly reduced cell viability (Figure 10A), which prevented the assessment of its potential specific effect on the TLR4/HIF‐1α pathway. The optimal concentrations of UDA, LA, and CA were determined to be 100, 50, and 150 μM, respectively, and these three compounds were used for further investigation. TGF‐β1 markedly inhibited cell viability, and simultaneous treatment with LA or CA partially restored it; in contrast, UDA had no effect on cell viability upon TGF‐β1 stimulation (Figure 10B). TGF‐β1 significantly increased TLR4 and HIF‐1α levels, which were inhibited by LA or CA (Figure 10C). However, UDA did not affect the levels of these proteins in TGF‐β1‐treated cells. This evidence suggests that the regulatory effect of QXLZY on the TLR4/HIF‐1α pathway may be related to LA and CA rather than UDA. TLR4 was overexpressed in HK‐2 cells to further confirm this hypothesis. Both LA and CA notably enhanced the viability of TGF‐β1‐treated cells, an effect that was partially reversed by the overexpression of TLR4 (Figure 10D). Moreover, both LA and CA significantly reduced TLR4 and HIF‐1α levels in TGF‐β1‐treated cells, but the overexpression of TLR4 partially counteracted this effect (Figure 10E).

## 4. Discussion

CKD is usually defined as impaired renal function lasting more than 3 months and is most commonly caused by diabetes and/or hypertension [1, 24]. Glomerulonephritis, infections, environmental exposures, and genetic risk factors may also increase CKD risk [24]. The 5/6 nephrectomy is a common modeling method for CKD. This model replicates human CKD characteristics, including increased Scr levels, proteinuria, tubulointerstitial injury, and fibrosis [25]. Similarly, we found that CKD rats showed decreased renal function, severe renal injury, and fibrosis, suggesting the successful establishment of the CKD model. To simulate renal tubular fibrosis in vitro, HK‐2 cells were induced by TGF‐β1. We found that TGF‐β1 significantly inhibited cell viability and promoted fibrosis in HK‐2 cells.

The pathogenesis of CKD is related to the loss of renal epithelial cells and the proliferation or recruitment of maladaptive cells (leukocytes and myofibroblasts) [26]. During this process, cell death regulatory pathways, such as ferroptosis, may contribute to kidney injury directly or by recruiting immune cells and inducing inflammation [26]. Wang et al. [27] found that tubular ferroptosis occurred in CKD mice, while fisetin improved CKD by inhibiting ACSL4‐mediated ferroptosis. Consistent with previous studies, we found that inflammation and oxidative stress levels were increased, and ferroptosis was activated in CKD models. This evidence suggests that ferroptosis may be a promising therapeutic target for CKD.

QXLZY exhibits significant benefits in CKD treatment. In a clinical trial, a modified QXLZY formula effectively controlled blood glucose, reduced proteinuria, alleviated oxidative stress and inflammatory responses, and delayed disease progression in patients with diabetic nephropathy [28]. QXLZY was found to improve the renal pathological changes in diabetic mice by restoring amino acid metabolism balance [29]. Consistent with these studies, we revealed that QXLZY significantly alleviated renal injury and fibrosis in CKD rats. Moreover, QXLZY partially restored cell viability and suppressed fibrosis in TGF‐β1‐stimulated HK‐2 cells. Notably, we found that QXLZY inhibited ferroptosis in CKD models. This effect was partially reversed by the ferroptosis inducer erastin, which also attenuated the protective effects of QXLZY on renal fibrosis. These findings indicate that ferroptosis is functionally involved in the regulation of renal fibrosis in CKD. However, the mechanism by which QXLZY regulates ferroptosis needs further exploration.

In the current study, we retrieved QXLZY, CKD‐related, and ferroptosis‐related targets from public databases. By intersecting these targets, 12 overlapping genes were obtained. GO analysis showed that these targets were mainly enriched in the positive regulation of chemokine production and miRNA transcription. Chemokines regulate leukocyte chemotaxis and tissue inflammation. Studies have shown that blocking chemokine CCL9 at CKD initiation promotes renal inflammation and fibrosis [30]. Furthermore, various miRNAs participate in renal fibrosis, promoting or inhibiting CKD progression [31]. KEGG analysis demonstrated that these overlapping genes were primarily enriched in the HIF‐1 pathway. Renal tissue hypoxia is related to CKD, and HIF is a type of transcription factor mediating hypoxia responses, playing an important role in renal fibrosis and contributing to CKD development [32]. Therefore, the genes (IL6, IFNG, TLR4, RTK, HIF1A, VEGF, and HMOX1) enriched in this pathway were considered potential key targets.

Based on the GSE66494 dataset, we found that EGFR, HIF1A, TLR4, and IFNG were significantly upregulated, while VEGFA was downregulated in CKD. Consistent with our findings, Cao et al. [33] found that EGFR expression is increased in fibrotic kidneys of both humans and mice, while selective EGFR deletion in fibroblasts/pericytes inhibited interstitial fibrosis. Furthermore, IFNG levels were notably increased in the kidneys of diabetic nephropathy rats but decreased after treatment with 22‐oxacalcitriol [34]. VEGFA maintains the renal microvascular system and is closely associated with interstitial fibrosis [35]. HIF‐1α levels are significantly upregulated in CKD rats, while Sanqi oral solution targets HIF‐1α to regulate PKM2‐activated glycolysis and improve renal fibrosis [36]. In the type 2 cardiorenal syndrome rat model, TLR4 and HIF‐1α levels are markedly increased, while Zhenwu decoction inhibits the TLR4/NF‐κB/HIF‐1α axis to alleviate renal fibrosis [37]. Therefore, we speculate that the TLR4/HIF‐1α pathway may be implicated in QXLZY treatment of CKD. Further experiments found that TLR4 and HIF‐1α levels were markedly upregulated in CKD models but were downregulated after QXLZY treatment. Additionally, overexpression of TLR4 notably increased HIF‐1α expression and altered the effects of QXLZY on fibrosis and ferroptosis in HK‐2 cells, which were reversed by the HIF‐1α inhibitor LW6. These results confirm that QXLZY inhibits ferroptosis through the TLR4/HIF‐1α pathway, thereby improving CKD. Furthermore, we found that TLR4 may primarily interact with UDA, LA, PA, and CA, which are mainly derived from Fuling, Huangqi, and Huangqin. Molecular docking further supported these findings by showing favorable binding affinities between these compounds and TLR4, suggesting potential direct interactions. In vitro experiments confirmed that LA and CA were bioactive components of QXLZY that regulate the TLR4/HIF‐1α pathway in CKD. Similarly, Gao et al. [38] demonstrated that organic acids are the main QXLZY components, and the pathways of QXLZY in treating diabetic nephropathy may involve glycolipid metabolism, oxidative stress, and inflammation.

This research has some limitations. First, the anti‐CKD mechanism of QLXZY has not been confirmed through in vivo gain‐of‐function experiments. Moreover, given the extensive cytotoxicity of PA in HK‐2 cells, we did not explore its effect on the TLR4/HIF‐1α pathway in CKD, and its potential specific effects should be investigated using lower, nontoxic doses. In addition, the results obtained in this study still require further clinical validation.

## 5. Conclusion

Collectively, our findings suggest that CKD is associated with activation of the TLR4/HIF‐1α pathway, which promotes renal tubular injury and fibrosis partly by regulating ferroptosis. QXLZY exerts renoprotective effects by suppressing the TLR4/HIF‐1α pathway, thereby inhibiting ferroptosis and subsequently attenuating renal fibrosis. Among the components of QXLZY, LA, and CA may be the key bioactive constituents contributing to these effects.

## Author Contributions

**Binqi Wang:** conceptualization, investigation, data curation, formal analysis, writing – original draft. **Bo Lin**: resources, formal analysis, software, obtaining funding, visualization, writing – original draft. **Danna Zheng**: resources, software, methodology. **Luxi Cao**: conceptualization, project administration, data curation, formal analysis, writing – review and editing.

## Funding

This study was supported by The Chinese Medical Science and Technology Project of Zhejiang Province (Grants 2024ZL008 and 2023ZR063).

## Disclosure

All authors read and approved the final manuscript.

## Ethics Statement

This study was approved by the Animal Ethical and Welfare Committee of Yanghzou University (Number 202505019), which was carried out in accordance with the U.K. Animals (Scientific Procedures) Act, 1986 and associated guidelines, EU Directive 2010/63/EU for animal experiments.

## Conflicts of Interest

The authors declare no conflicts of interest.

## Supporting Information

Additional supporting information can be found online in the Supporting Information section.

## Supporting information


**Supporting Information** Table S1: The mobile phase conditions for HPLC‐MS analysis. Figure S1: HPLC‐MS analysis of QXLZY.

## Data Availability

The data that support the findings of this study are available from the corresponding author upon special request.
