# Supplementary material for: Qingxin Lianzi Yin Improves Chronic Kidney Disease by Targeting Ferroptosis via the TLR4/HIF‐1α Pathway
Source: Mediators Inflamm. 2026 Jun 5;2026:7440641. doi: 10.1155/mi/7440641 (PMC13238268; doi:10.1155/mi/7440641)
Supplement: Supplementary file 1 — Supporting Information Table S1: The mobile phase conditions for HPLC‐MS analysis. Figure S1: HPLC‐MS analysis of QXLZY. [file MI-2026-7440641-s001.docx]

**Qingxin Lianzi Yin improves** **chronic kidney disease by targeting ferroptosis via the** **TLR4/HIF-1α pathway**

**Running title:** QXLZY improves CKD

Binqi Wang^1#^, Bo Lin^1#^, Danna Zheng^1^, Luxi Cao^1*^

^1^Urology & Nephrology Center, Department of Nephrology, Zhejiang Provincial People's Hospital (Affiliated People's Hospital, Hangzhou Medical College), Hangzhou, Zhejiang, China

# Binqi Wang and Bo Lin contributed equally to this study.

**^*^Corresponding author:** Luxi Cao

Address: Urology & Nephrology Center, Department of Nephrology, Zhejiang Provincial People's Hospital (Affiliated People's Hospital, Hangzhou Medical College), Hangzhou 310000, Zhejiang, China

Email: [lucycao2019@163.com](mailto:lucycao2019@163.com) and caoluxi@hmc.edu.cn

**Supplementary materials include 1 table and 1 figure.**

**Table S1** The mobile phase conditions for HPLC-MS analysis

| Time (min) | | Mobile phase A (% v/v) | | Mobile phase B (% v/v) |
| --- | --- | --- | --- | --- |
| 0.0 | 98 | | 2 | |
| 5.0 | 98 | | 2 | |
| 8.0 | 80 | | 20 | |
| 45.0 | 45 | | 55 | |
| 52.0 | 0 | | 100 | |
| 55.0 | 0 | | 100 | |
| 55.1 | 98 | | 2 | |
| 58.0 | 98 | | 2 | |


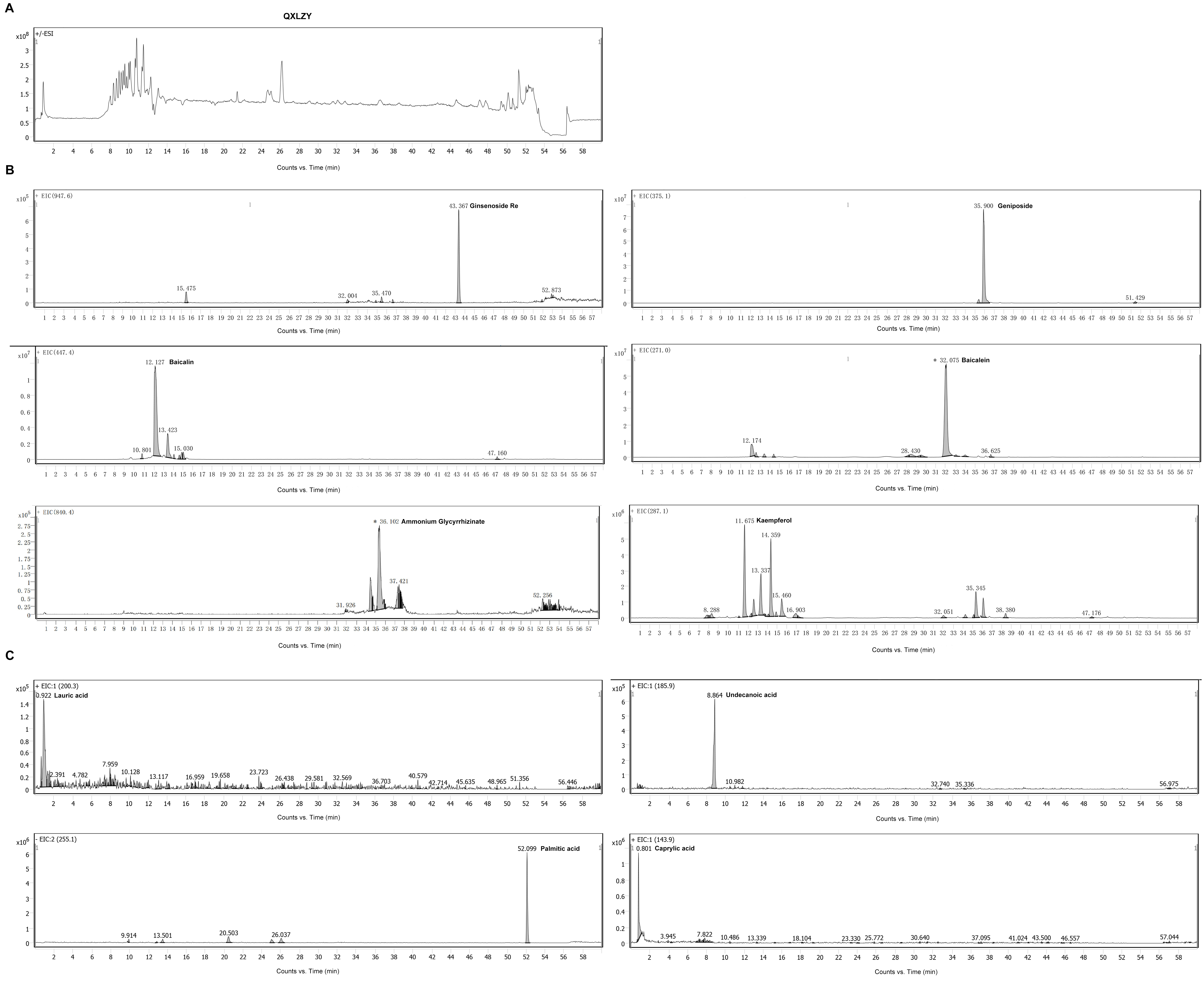


**Figure S1** HPLC-MS analysis of QXLZY. (A) Total ion chromatogram of QXLZY. (B) Extracted ion chromatograms of ginsenoside Re (m/z 947.6), geniposide (m/z 375.1), baicalin (m/z 447.4), ammonium glycyrrhizinate (m/z 840.4), kaempferol (m/z 287.1), and baicalein (m/z 271.0) in the QXLZY sample, representing the major constituents of the formula. (C) Extracted ion chromatograms of lauric acid (m/z 200.3), undecanoic acid (m/z 185.9), palmitic acid (m/z 255.1), and caprylic acid (m/z 143.9) in the QXLZY sample, which were identified as key active components based on network pharmacology analysis. m/z, mass-to-charge ratio.
